# Electronic medical record implementation in a large healthcare system from a leadership perspective

**DOI:** 10.1186/s12911-022-01801-0

**Published:** 2022-03-15

**Authors:** Yaseen M Arabi, Abdullah Ali Al Ghamdi, Mohamed Al-Moamary, Abdullah Al Mutrafy, Raed H. AlHazme, Bandar Abdulmohsen Al Knawy

**Affiliations:** 1 College of Medicine, King Saud Bin Abdulaziz University for Health Sciences, Riyadh, Saudi Arabia; 2 King Abdullah International Medical Research Center, Riyadh, Saudi Arabia; 3 Intensive Care Department, King Abdulaziz Medical City, Riyadh, Saudi Arabia; 4 Clinical Affairs, Family Medicine and Primary Healthcare, King Abdulaziz Medical City, Riyadh, Saudi Arabia; 5 Development and Quality Management, King Saud Bin Abdulaziz University for Health Sciences, Riyadh, Saudi Arabia; 6 King Abdulaziz Medical City, Riyadh, Saudi Arabia; 7 King Abdullah Specialized Children’s Hospital, Riyadh, Saudi Arabia; 8 Ministry of National Guard Health Affairs, Riyadh, Saudi Arabia; 9 College of Public Health and Health Informatics, King Saud Bin Abdulaziz University for Health Sciences, Riyadh, Saudi Arabia; 10 Information Technology Department, King Abdulaziz Medical City, Riyadh, Saudi Arabia; 11 College of Osteopathic Medicine, Nova Southeastern University, Fort Lauderdale, FL USA; 12 King Saud Bin Abdulaziz University for Health Sciences, Riyadh, Saudi Arabia

**Keywords:** Leadership, Electronic medical record, Models of change, Leadership approach

## Abstract

**Background:**

Information on the use of change management models to guide electronic medical records (EMR) implementation is limited. This case study describes the leadership aspects of a large-scale EMR implementation using Kotter’s change management model.

**Methods:**

This case study presents the experience in implementing a new EMR system from the leadership perspective at King Abdulaziz Medical City, a large tertiary care hospital in Riyadh, Kingdom of Saudi Arabia. We described the process of implementation and outlined the challenges and opportunities, throughout the journey from the pre-implementation to the post-implementation phases.

**Results:**

We described the corresponding actions to the eight domains of Kotter’s change management model: creating a sense of urgency, building the guiding team, developing a change vision and strategy, understanding and buy-in, removing obstacles, creating short-term wins, building on the change and anchoring the changes in corporate culture.

**Conclusions:**

The case study highlights that EMR implementation is not a pure information technology project but rather is a technical-based complex social adaptive project that requires a specific set of leadership competencies that are central to its success. It demonstrates that change management models might be useful for large-scale EMR implementation.

## Background

Driven by data demonstrating positive effects of electronic medical records (EMR) on clinical and operational outcomes, there has been an increased implementation of EMRs in healthcare systems [1, 2]. In the USA, the Meaningful Use (MU) program was established to facilitate nationwide adoption of EMRs, and resulted in a steep increase in EMR implementation, leading to > 90% of hospitals using a government-certified EMR [3]. Globally, EMR implementation is also increasing, albeit to varying degrees across different countries [5, 6]. The widespread of EMR implementation has exposed several challenges [3]. One of the major challenges is that EMR implementation can be ineffective, resulting in uninstallation after implementation, substantial delays in implementation and cost overruns or failure to use EMR to full potential [7, 8] A fundamental reason for ineffective EMR implementation is related to treating EMR implementation as a pure information technology (IT) project, instead of being a business process transformation, which involves IT components [9, 10]. Studies have highlighted the importance of the interaction of the social features of a healthcare environment with the technical features of the EMR during implementation [9, 10]. It has been suggested that such interaction could be managed effectively by establishing strong leadership, using project management techniques, ensuring efficient communication and establishing standards and training [9, 10]. However, there is limited data in health IT implementation literature on the use of a change management model in addressing the social features of the healthcare environment to ensure effective EMR implementation. The use of a change management model would logically focus on the people side of the change; and would largely depend on the organizational culture, context, and resources [10].

The objective of this paper was to describe a case study of the leadership aspects of EMR implementation utilizing Kotter’s change management model [11]. In addition, we provided post-hoc reflections on the implementation experience utilizing Lukas’s and Lewin’s change management models [12, 13]. The presented case study may serve as an example for healthcare leaders who envision the large-scale implementation of EMR in their organizations.

## Methods

### Setting

This case study presents the experience at King Abdulaziz Medical City, Riyadh (which will be referred to as KAMC-R), a large tertiary care hospital, in the Kingdom of Saudi Arabia and is the largest hospital among the five medical campuses in the healthcare system of the Ministry of National Guard Health Affairs (MNGHA). The institution provides integrated primary, secondary, and tertiary care with over 100 training programs in all clinical disciplines. At the time of EMR implementation, it had 17 medical and 9 operational departments. The hospital had a capacity of approximately 1700 operational beds with a large ambulatory care center that included different specialty outpatient clinics and 54 satellite sites for primary care that provided approximately 1,400,000 outpatient visits.

King Abdullah Specialized Children Hospital (KASCH) was a new building within the same medical campus that opened during the EMR implementation project. This facility started operating with the new system, which served as a proof of concept for the more extensive later big-bang implementation at KAMC-R (the subject of our paper); this early implementation will not be discussed in this paper. The hospital system’s employees were from varying backgrounds and nationalities. English was the official written language for medical records. Before the EMR implementation, KAMC-R used paper charts for documenting clinical encounters but had an electronic system for laboratory and radiology reports and computerized physician order entry (CPOE). However, all primary health care centers were not electronically connected and carried out their work entirely on paper.

The new EMR system (BESTCare system, Saudi-Korean Health Informatics Company, Riyadh) was an integrated system that included patient medical record, computerized physician order entry (CPOE), clinical decision support system (CDSS), closed-loop medication administration (CLMA), clinical data warehouse (CDW), health information exchange (HIE), and disaster recovery (DR) [14, 15]. The system was supplemented with a big data warehouse and healthcare business intelligence tools. The system source code and Intellectual Property (IP) were owned by the MNGHA, which meant that its further development to meet the needs of the patients and the organization’s strategic objectives were highly achievable. An EMR implementation committee was formed in October 2014; fifteen months before the anticipated implementation date, and was chaired by a physician from the hospital medical administration. The committee was composed of members representing different disciplines from the hospital medical administration (chair), medical departments (3 members), other clinical and operational departments (3 members) and information technology departments (5 members).

### Measurements

Kotter’s change management model was articulated as a change management model for the current EMR implementation, based on previous experience in other projects by two of the authors [11]. The philosophy of this paper was a narrative description using a social constructivist approach given its relative interpretations. The narrative description was the personal recollection of the project facts and review of the administrative records (meeting minutes, meeting presentations, memoranda and time tables) by the authors of this paper, who had been engaged in the EMR implementation, either as a chair or members of the EMR implementation committee (YA, AG) or as healthcare system leadership (MA, AM, RH, BK). There were no additional interviews or recorded meetings.

In this report, we described the process of EMR implementation and outlined the challenges and opportunities, throughout the journey from the pre-implementation to the post-implementation phases. We described the corresponding actions to the eight domains of Kotter’s change management model: creating a sense of urgency, building the guiding team, developing a change vision and strategy, understanding and buy-in, removing obstacles, creating short-term wins, building on the change and anchoring the changes in corporate culture [11]. In addition, we provided post-hoc reflections on the implementation experience utilizing Lukas’s and Lewin’s change management models [12, 13].

To provide quantitative outcome measures of the implementation of EMR, we supplemented this report with aggregate data on selected indicators, obtained from administrative records. We reported the number of daily EMR transactions (laboratory, radiology and medication transactions and form creation) in the first week after implementation and 4 years later. We also reported the activity of the command center during the implementation by documenting the number of calls to the command center (described later) during the 2-week implementation. As a balancing measure, we documented the number of reported medication errors during the implementation phase due to the transition and we reported healthcare worker satisfaction (using a locally developed satisfaction tool) after the implementation. We also listed selected examples of clinical and operational improvements and the resilience of the healthcare system during the coronavirus disease 2019 (COVID-19) pandemic. Because of the nature of aggregate data, statistical comparisons were not possible. The study was approved by the Institutional Board Review of the Ministry of the National Guard Health Affairs, Riyadh, Saudi Arabia.

## Results

### Understanding the context: the expected and unexpected challenge

The MNGHA had made a significant investment in the new EMR. It had hoped that the multi-level implementation would cover inpatient services, outpatient clinics and primary health centers that were distributed over a wide geographic area in Saudi Arabia. The premise was to transform the system, once and for all, to become completely paperless and to integrate the different components of the system. Before the implementation of the full-suite EMR, the hospital utilized a hybrid system in which an electronic system was used to support the paper-based medical records and enabled reviewing results and entering essential patient data.

Over ten years, the MNGHA health system had grown from two middle size hospitals in the 1990s to a multi-dimension healthcare institute with five medical campuses, a research center (King Abdullah International Medical Research Center, KAIMRC), and three university campuses (King Saud bin Abdulaziz University for Health Sciences, KSAU-HS). Such vast and rapid growth raised challenges in the utilization of the existing hybrid medical records to provide safe patient care. Different solutions were available; however, the organization leadership proceeded to an innovative approach, in which it had a joint venture with the University of Seoul, South Korea, to further develop and internationalize an existing EMR, named BESTCare, and customize it to meet MNGHA’s needs. The leadership was looking forward to a successful implementation in the largest hospital in the MNGHA system (KAMC-R) to serve as a model for implementation in the remaining four campuses of the healthcare system. The joint work progressed reasonably well since the commencement of the project in 2014.

However, in the summer of 2015, the unexpected happened when KAMC-R was affected by a major outbreak of the Middle East Respiratory Syndrome coronavirus (MERS-CoV) [16]. This infection was recognized as a severe respiratory viral infection that was easily transmitted within healthcare settings and was associated with high mortality [17]. The hospital had to close most of its services for almost two months to avoid further progression of the outbreak, protect patients and healthcare workers, and upgrade infection prevention measures [18]. As a result, and for almost four months, much of the preparatory work to implement the long-waited EMR slowed down. However, in the aftermath of the outbreak and with the new leadership of the hospital management and with many challenges during the process of re-opening the hospital, the organization found itself only six weeks from the non-changeable implementation date on the 20^th^ of January 2016. This date was established as a part of the timeline for implementing the EMR in the five campuses in the healthcare system and required a combined work of multiple technical parties. The hospital was also up to another fast-approaching challenge; it had to go through re-accreditation by the Joint Commission International (JCI) in March 2016 that included new standards for being accredited as an academic institute for the first time. It was obvious that the hospital was approaching an enormous task that had put the whole process at stake. Facing these challenges, the MNGHA leadership appointed a new clinical chair for the EMR implementation committee shortly before the planned EMR go-Live. It was soon recognized that the only way forward to overcome the emergent situation was to mobilize the whole organization to meet this critical strategic target by urgently re-promoting the organization's vision, building a new team, and relying on a task-oriented transactional approach.

### Moving the giants

The overwhelming impact and the scars produced by the recent MERS-CoV outbreak, along with the pressing need to meet the approaching deadline for EMR implementation, created the urgency for an intense mobilization of all human and non-human resources. The EMR implementation committee realized its central role in leading the change. The prevailing theme of the implementation process was an engagement at all levels: from the frontline clinicians and other healthcare workers to the leadership of the healthcare system. In Table 1, we describe the leadership aspects of the implementation process using Kotter’s change management model [11].

**Table 1:** Examples from the case study of implementation of electronic medical record (EMR) of the corresponding actions to the eight domains of Kotter’s change management model

Domains of Kotter’s model	Corresponding actions
Creating a sense of urgency	Creating readiness assessment tool that captured critical actions needed for implementation and could be used across the different clinical and non-clinical departments Sharing the readiness assessment tool with hospital and department leaders frequently to tract preparedness, stimulate peer-feedback and increase progress
Building the guiding team	Transforming the EMR implementation committee to the active and engaged mode Forming departmental implementation teams to drive change from within
Developing a change vision and strategy	Integrating the continuum of care from the community to specialized care in a seamless fashion Standardization of care through protocols, order sets, and clinical pathways Setting the basis for a data-driven organization Creating communities of practice Implementing a multidisciplinary approach in the new workflows The emphasis on safe implementation for patients Recognizing the highest potential threats (e.g. errors in transferring data on drug allergies) and having multiple layers of protection Emphasizing staff-friendly process
Understanding and buy-in (Communicate the vision)	Official communications Emails and text messages Pamphlets and posters Screen savers Digital signage system Presentations and forums Huddles Immediate supervisors and super users
Removing obstacles	Addressing resistance at departmental and individual levels Understanding the emotions of people Engagement in the process of implementation Training Taking input seriously Peer feedback Converting resistant individuals to strong advocates Rarely executive interventions
Creating short term wins	Winning key people at executive level and clinical chairs Creating easy training sessions Using tools for feedback Simulation Daily briefing in the week preceding the implementation Emphasizing safe process: for example: allergies/medication Command center Multiple testing Support at go-live
Building on change (consolidating gains)	Daily debriefing Sharing data from the command center with frontline staff Having multiple rounds of training Emphasizing the benefits of the new system
Anchoring the change in corporate culture	Emphasizing how the organization had altered incoherent practices Demonstrating the value of working together (communities of practice) Emphasizing that patient safety was at the core value of go-live Demonstrating that post-implementation issues were much lower than expected Development of EMR enhancement committee

**Fig. 1:**
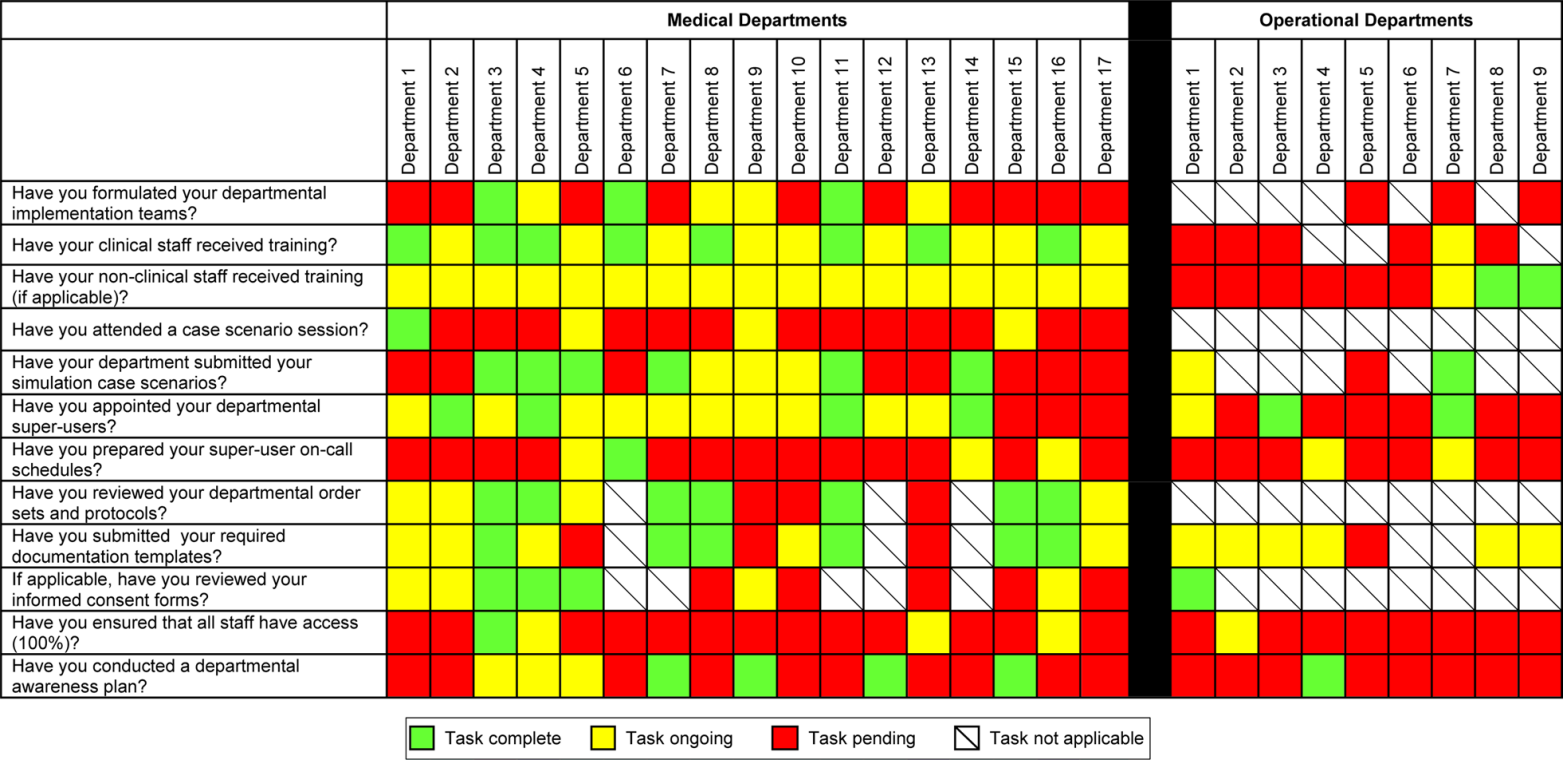
Readiness assessment tool used during the electronic medical record (EMR) implementation process. The tool captured critical tasks needed for implementation and was used across different medical and operational departments. The tool was shared with hospital leaders and department leadership frequently to track preparedness, peer-feedback, and expedite progress. Completed tasks were color-coded green, tasks in progress were color-coded yellow, and deficient tasks were color-coded red. When more than 95% of project tasks were green color-coded in any unit, that unit was considered ready. Overall, all essential project tasks were green color-coded at the time of the big bang implementation (go-live)

To “*create a sense of urgency”*, the EMR implementation committee developed a readiness assessment tool that captured critical actions needed for implementation across different medical and operational departments (Fig. 1). The readiness assessment tool was context-specific and was developed by consensus by the EMR implementation committee. It was shared with hospital leaders and department leadership frequently to track preparedness, stimulate peer-feedback, and increase progress. The EMR implementation committee requested all departments to form departmental implementation teams to serve as departmental “*guiding teams”* and ensure full engagement of staff who were familiar with day-to-day workflow operations. Each department was requested to nominate “superusers” who underwent specialized training on the EMR system; these superusers, in turn, served as the resource for training and support at the departmental level. A structured scenario-based training program was formulated. A designated area was assigned as a training laboratory for all hospital staff and was manned by clinical, operational and technical staff. Each department was charged with ensuring that all clinical and operational staff received the mandatory training sessions on the new EMR. This required that departments would need to plan allowing sufficient time for the required staff during the 6-week pre-implementation period. With a large and complex hospital multi-departmental system, transforming the clinical protocols, documentation templates and informed consents from paper to electronic forms was not an easy task; and the only effective way was to charge each departmental implementation team with working closely with IT teams to ensure completion of all required protocols before the go-live. Finally, each department was charged by ensuring that all staff members had received the electronic access credentials and that a departmental awareness plan was put in place. To track this complex task list, the readiness assessment tool was updated and submitted weekly to ensure compliance of all departments. Completed tasks were color-coded green, tasks in progress were color-coded yellow, and deficient tasks were color-coded red. When more than 95% of project tasks were green color-coded in any unit, that unit was considered ready. Overall, all essential project tasks were green color-coded at the time of the big bang implementation (go-live).

The EMR implementation committee was responsible for developing a “*change vision and strategy*”, which focused on integrating the continuum of care from the community to specialized care in a seamless fashion. Moreover, it oversaw the standardization of care through protocols, order sets and clinical pathways, setting the basis for a data-driven organization, creating communities of practice, implementing a multidisciplinary approach in the new workflows and emphasizing staff-friendly processes.

To gain “*understanding and buy-in****”***, the EMR implementation committee maintained open communication with the organization leadership, through frequent presentations and reports; the leadership engagement and support were central to the success of the project. The EMR implementation committee launched a hospital-wide awareness campaign using official communications, emails and text messages, pamphlets and posters, computer screen savers, digital signage system, presentations, forums and huddles and through immediate supervisors and superusers with the overarching goal of keeping the staff abreast of ongoing activities, plans, and requirements.

A vital task of the committee was to “*remove obstacles”,* particularly mitigating and overcoming resistance at departmental and individual levels by engaging staff in the process of implementation and training, and by taking input and peer feedback seriously. Such plans were successful in converting resistant individuals to strong advocates; executive interventions were rarely required to address resistance.

To keep the momentum, it was essential to “*create short-term wins*”. Winning key people at the executive level or clinical chairs was essential to give such a message. Creating effective training sessions to demonstrate that implementing EMR would likely facilitate care, and using tools for feedback and emphasizing a safe environment also helped in reducing anxiety and maintaining engagement. Moreover, a full vacated hospital ward was used for pre-implementation simulation of patient care from the community to the specialized services. It was a great opportunity not only to test the technical aspects of the system but also to demonstrate to all staff that the whole project was going in the right direction. The visibility and support of the top hospital leadership delivered another critical message for building confidence. In the week preceding the planned go-live, the EMR implementation committee held daily open briefings in the hospital's large auditorium, with the attendance of all involved hospital leadership. All plans were reviewed with a particular emphasis on the safe implementation process. For example, the committee identified medication errors in transferring data on drug allergies as one of the highest potential threats that might occur during implementation. As such, the committee developed multiple layers of protection (double-checks by a physician, a nurse and a pharmacist, and auditing by the quality department). A command center was established to guide the overall process and to be the resource for any inquiries that might arise during the implementation process.

The go-live day for in-patient services was planned on a weekend, but it was not like other weekends. The hospital leadership, clinical chairs, and consultants were all physically on-site. The implementation procedures during go-live were followed as planned, and the transition to the new EMR was uneventful. All patient care and administrative processes were executed smoothly with no significant interruptions. Forty-eight hours later, the outpatient and primary health care go-live was launched. On that day, a limited technical issue, called backend resource saturation, occurred in the outpatient and primary health care setting due to high traffic and led to initial launch failure. The already prepared contingency plan that included reverting to paper records was immediately activated, and all services resorted; no patient care or administrative process was affected. The technical issue was resolved within the same day, and the system ran smoothly thereafter. Within three days, the whole hospital system with all its satellites was running smoothly on the new EMR system.

Next, it was essential to “build on change and consolidate gains”. Following implementation, the EMR implementation committee held daily debriefing for a week. The data from the command center confirmed the progressive adoption of the system by all staff within a short period; these data were used to provide feedback to staff to provide further confidence in the system. It was reassuring to all staff to hear that there were no significant reported patient safety incidents related to the EMR system and that there were no significant difficulties reported that could not be addressed by the command center (Table 2). All issues that were reported to the command center were about further training and workflow, and all were resolved immediately with feedback provided to the related staff. It was also reassuring to hear the staff satisfaction with the system; in fact, there was no resignation or transfer from one service to another due to the new system.

**Table 2:** Hospital characteristics and EMR implementation key statistics

*Hospital characteristics*		
Number of departments	17 medical and 9 operational department	
Number of primary healthcare centers	12 large centers, and 15 small satellites	

*EMR transactions (per day)*		
	EMR Go-Live average daily 24–28 January 2016	03 December 2019
Appointments	14,643	21,410
Laboratory requests	8662	10,549
Radiology requests	1385	1,271
Medication requests	7162	24,152
Form creation (documentation)	7227	16,586
*Command center*		
Numbers of calls to command center during the 2-week implementation	Total number of calls was 3,587 including 2,654 for technical issues and 933 for content help inquiries	
Number of significant medication errors during the implementation due to the transition	None	
Healthcare worker satisfaction-2018	Very Satisfied—24.1% Satisfied—46.9% Neutral—21.9% Dissatisfied—5.7% Very Dissatisfied—1.4%	
***Examples of clinical and operational improvement***		
Patient satisfaction	Composite patient satisfaction regarding access to care among patients attending the employee health center increased from 4/10 prior to EMR implementation to 9/10 in 2018	
Example of Clinical outcomes	Improved glycemic control of the uncontrolled type 2 diabetic patients by 27% in 2019 compared to 2018 in a population of 11,495 poorly controlled diabetic patients	
Efficiency	Waiting time in primary health clinics from check-in to the actual appointment averaged in hours prior to EMR implementation and decreased after the system in 2019 to 3 min and 22 s The appointment capacity improved from an average of 2600 per day to 4000 per day	

Sustainability required “anchoring the change in corporate culture”. As the KAMC-R has adopted the system since day one of the go-live, the system became part of the organization's culture. The command center was maintained active for the first two weeks after implementation and then it was closed because no further issues were reported. This indicated how the implementation was efficient and the system was adopted quickly. The EMR was associated with a high rating of patient and staff satisfaction and had helped in improving processes of care and efficiency, an example of which is provided in Table 2. The further development of the EMR Enhancement Committee to continue with system development gave another message about the stability of the system.

Furthermore, the KAMC-R successfully passed the JCI accreditation in March 2016, only two months after EMR implementation, with almost 97% compliance with the required standards and received the certificate as an Academic Medical Center Hospital Program. Over the following year, the EMR was implemented in the other four MNGHA campuses in Jeddah, Madinah, Dammam and Alhasa. Now, six years after the implementation, the EMR implementation has resulted in the integration of care across widely distributed primary healthcare clinics across the Kingdom with secondary and tertiary care. It has led to the standardization of a large number of processes of care using order sets and protocols. As an example of how EMR facilitated care, glycemic control of the uncontrolled type 2 diabetic patients improved by 27% in 2019 compared to 2018 in a population of 11,495 poorly controlled diabetic patients in primary healthcare centers. (Table 2) The EMR has facilitated efficiency in primary healthcare centers, patient waiting time from check-in to the actual appointments averaged in hours, and was reduced after EMR implementation to less than four minutes in 2019. The appointment capacity improved from an average of 2600 per day to 4000 per day. In 2019, the hospital received the Healthcare Information and Management Systems Society (HIMSS) Outpatient-EMRAM Stage 7 accreditation (https://www.ngha.med.sa/English/MediaCenter/News/Pages/MMXIXIVI.aspx), and in 2021 received the HIMSS Adoption Model for Analytics Maturity (AMAM) Stage 7 accreditation (https://www.healthcareitnews.com/news/emea/two-medical-groups-saudi-arabia-achieve-stage-7-digital-maturity). The EMR was also used to build an electronic alert system for sepsis that has been implemented throughout the 5 medical campuses, as part of a stepped-wedge randomized trial that included more than 60,000 hospitalized patients [19, 20] Furthermore, the EMR has facilitated a quick efficient transformation during the COVID-19 pandemic with the implementation of new screening tools, tracking of patients, and monitoring of their medical care [21–23]. It also allowed rapid implementation of the concepts of virtual clinics and telehealth. Such an experience reflects the contribution of the EMR to the resilience of the healthcare system [21–23].

### Lukas’s and Lewin’s change management models

We also reviewed the experience using other models. Lukas’s change management model, proposed for organizational transformation in healthcare systems, has five elements [12]. *“Impetus to transform”* in our case was reflected by the urgency to implement the EMR. *“Leadership commitment”* to quality was reflected by a clear vision to transform the organization to use a full-suite EMR system. *"Improvement initiatives that actively engage staff in problem-solving”* were evident by the engagement of departmental teams and staff in the change process. *“Alignment to achieve consistency of organization goals with resource allocation and actions at all levels of the organization”* was demonstrated by the coordinated work to mobilize the resources for implementation. *“Integration to bridge traditional intra-organizational boundaries”* among individual components was exemplified by the multidisciplinary approach and by the integration of the spectrum of the multi-layer healthcare system from primary to tertiary. These elements drove change by affecting the components of the complex healthcare organization and by dealing with the mission, vision, and strategies; culture; operational processes and infrastructure such as IT and human resources [12].

We also reviewed the experience using Lewin’s change management model, which consists of three steps: unfreeze, change, and refreeze [13]. One may consider the first three steps in Kotter’s change management model: “creating a sense of urgency, building the guiding team and developing a change vision and strategy” to correspond with the “unfreeze” step in Lewin’s change management model, and the “understanding and buy-in and removing obstacles” to correspond with the “change” step and finally, “creating short-term wins, building on the change and anchoring the changes in corporate culture” to correspond with the “refreeze” step [11–13].

## Discussion

In this paper, we described a successful EMR implementation from a leadership perspective, using Kotter’s change management model. This case study highlights that EMR implementation, and perhaps large-scale IT projects in general, are not pure IT projects, but IT-based complex social adaptive projects that require a specific set of leadership competencies. These include clear vision, guiding leadership, and collaboration between IT and healthcare workers. Unit-based teams are also essential to execute the project at smaller (micro) levels. This project has proven this logical argument. Such a massive project could not have been executed without a theoretical underpinning of the leadership approach (especially change management). In our example, the project was implemented as a big bang approach. However, we argue that it was not only the type (i.e., big bang, waterfall, etc.) or the scale of implementation that mattered, but as importantly, it was the leadership commitment and the wide-scale engagement of staff across all levels. As emphasized by Kotter, it is essential to note that the domains of work require continuing attention through a change process and should not be approached in a linear "method" for making change. For example, attention to some of these domains, such as “understanding and buy-in”, “removing obstacles” and “creating short-term wins”, was required throughout the implementation process. While we found that all domains of Kotter’s change management model were necessary, we noted that “creating a sense of urgency”, “understanding and buy-in”, “removing obstacles” and “creating short term wins” were of particular relevance to our culture that values urgency, communication from authority and quick wins. The relative importance of Kotter’s domains may vary across different settings and cultures.

It has been suggested that two framework types could guide IT implementation: technology adoption models and implementation science models [24]. Technology adoption models use leadership or change management concepts and focus mainly on how the end-users adopt technology, whereas implementation science models describe methods and interventions to implement evidence-based practice [24]. Although our approach is an example of the use of a technology adoption model, our report highlights that the change management aspects cannot be separated from the technical implementation. Kotter’s change management model has a long-standing track record and has been considered to be appropriate for a variety of large-scale implementations, including the implementation of EMR in various settings [25–27]. This has been highlighted in a qualitative study that included 47 physicians and 35 administrators who had participated in successful EMR system implementations in the USA [28]. The study identified Kotter’s framework as a good example of a change management model that appeared to address the challenges of EMR implementation [28]. There are several implementation science models for information technology that have been described, but data about their use in large-scale implementation are limited [29–31].

This case study is not without limitations. It is descriptive and cannot account for all factors that are critical to the success of such projects like the influence of organizational culture in such a massive change, motivation and human learning and coping skills. The case study did not address the long-term commitment of the stakeholders to use and develop the EMR; this could serve as an area of future research in leadership and implementation science. We reported several quantitative indicators, as obtained from administrative records, to supplement the main report on leadership. However, because of the nature of aggregate data, statistical comparisons were not possible. Therefore, these data should be considered exploratory. Additionally, these findings might represent a glimpse of what might take a longer time to manifest as outcomes.

## Conclusions

This case study highlights that EMR implementation is not a pure IT project but an IT-based complex social adaptive project requiring a specific set of leadership competencies that are central to its success. It contributes to the literature indicating that the change management models might be useful for large-scale EMR implementation.

## Data Availability

Data sharing does not apply to this article as no datasets were generated or analyzed during the current study.
